# Kernel-based Joint Feature Selection and Max-Margin Classification for Early Diagnosis of Parkinson’s Disease

**DOI:** 10.1038/srep41069

**Published:** 2017-01-25

**Authors:** Ehsan Adeli, Guorong Wu, Behrouz Saghafi, Le An, Feng Shi, Dinggang Shen

**Affiliations:** 1 Biomedical Research Imaging Center (BRIC) and Department of Radiology, University of North Carolina at Chapel Hill, NC, 27599, USA; 2 Cedars-Sinai, Biomedical Imaging Research Institute, 8700 Beverly Blvd., Los Angeles, CA 90048, USA; 3 Department of Brain and Cognitive Engineering, Korea University, Seoul 02841, Republic of Korea

## Abstract

Feature selection methods usually select the most compact and relevant set of features based on their contribution to a linear regression model. Thus, these features might not be the best for a non-linear classifier. This is especially crucial for the tasks, in which the performance is heavily dependent on the feature selection techniques, like the diagnosis of neurodegenerative diseases. Parkinson’s disease (PD) is one of the most common neurodegenerative disorders, which progresses slowly while affects the quality of life dramatically. In this paper, we use the data acquired from multi-modal neuroimaging data to diagnose PD by investigating the brain regions, known to be affected at the early stages. We propose a joint kernel-based feature selection and classification framework. Unlike conventional feature selection techniques that select features based on their performance in the original input feature space, we select features that best benefit the classification scheme in the kernel space. We further propose kernel functions, specifically designed for our non-negative feature types. We use MRI and SPECT data of 538 subjects from the PPMI database, and obtain a diagnosis accuracy of 97.5%, which outperforms all baseline and state-of-the-art methods.

Diagnosis of neurodegenerative diseases based on neuroimaging data has remarkably improved patient treatment strategies. However, early diagnosis is a very challenging task, due to various sources of inter- and intra-subject variabilities, within and between different groups, as well as the induced deviations due to image acquisition and preprocessing procedures. In addition, the need for the identification of biomarkers for the disease further intensifies these challenges. Imaging biomarkers are essentially a set of image-based features, and therefore, feature selection techniques are heavily researched (*e.g*., refs 1 and 2) and have yielded eminent achievements.

Many previous works in the literature have investigated both linear and non-linear classification models for the task of disease diagnosis using neuroimaging data^1^,^3^,^4^. One of the closures in almost all these works is that linear and non-linear models both obtain similar performances for disease diagnosis based on neuroimaging data. However, a very crucial step in these works is the feature selection procedure, which is often overlooked. Current methods use either unsupervised (*e.g*., Fisher score^5^) or supervised feature selection (*e.g*., sparse feature selection^6^, or minimum-redundancy maximum-relevancy^7^) techniques. When a linear feature selection technique is used, the selected features would be most correlated linearly with the class labels (maximum relevance), while being least correlated with each other (minimum redundancy), in the *input feature space*. Nonetheless, this *linearly* selected feature set would not be the best for a *non-linear* classifier, which would map the input feature space to a higher dimensional space (denoted as kernel space). Accordingly, for the case of non-linear classification, it would be better to select the features that result in the best performance in the non-linear kernel space, rather than the original input feature space, illustrated in Fig. 1.

On the other hand, the choice of the mapping function to project the input feature space to a higher dimensional space is itself challenging, and dependent on the application and its associated data types. These non-linear projections are often modeled using kernel functions^8^. In this paper, we propose a new strategy to select the features that can best construct a classifier in the kernel space, as opposed to the conventional methods that select features only in the original input feature space. In this way, if we use a non-linear kernel, the selected features work best in the non-linear kernel space, or if a linear kernel is used, those features will be best for a linear classifier. To take advantage of more kernel types, we define a new kernel which interweaves different linear and non-linear kernels, so that takes advantage of both types of models. We apply our method to the problem of PD diagnosis using the Parkinson’s Progression Markers Initiative (PPMI) database^9^, available at http://www.ppmi-info.org/data. We use densities in Regions-of-Interest (ROIs) as features from Magnetic Resonance Imaging (MRI) and Single-Photon Emission Computed Tomography (SPECT) modalities. These features are volumetric non-negative values, and therefore we incorporate such information in our proposed kernel function.

It is important to note that, based on various studies in the literature^10^,^11^, usually non-linear classifiers can be better fit, if the data spans in a lower-dimensional space. Feature selection methods are often used in many neuroimaging applications^12^,^13^,^14^, leading to a limited number of selected features, and therefore we are dealing with a low-dimensional space for the classification task. This observation motivates us to specifically investigate non-linear models for classifying these data. But as mentioned above, in this paper we argue that if one chooses a non-linear classifier, the feature selector should be designed accordingly, and hence, we develop a joint model. However, it is not appropriate to assume that a non-linear model is always better than a linear model (or the native feature space). As a result, we formulate the framework such that both linear and non-linear kernels can be utilized and the model selects which operates best on the specific dataset.

Note that current practical diagnosis of PD mainly depends on the clinical symptoms. Clinically, PD is characterized by tremor at rest, rigidity, akinesia (or bradykinesia), postural instability, flexed posture and freezing (motor blocks) including also non-motor symptoms such as cognitive and psychiatric impairment^15^. PD symptoms start to appear with the loss of neurotransmitters in the brain, particularly dopamine. As discussed in the literature^16^,^17^,^18^,^19^,^20^, neuroimaging techniques can be successfully utilized for early diagnosis of PD. For instance, SPECT imaging is usually considered for the diagnosis of PD^18^,^19^, and MRI is often employed for the differential diagnosis of PD syndromes^19^,^21^,^22^, as well as the analysis of the structural changes in PD patients^16^,^23^,^24^. Recently, many researchers started using machine learning and data-driven analysis methodologies for disease diagnosis purposes, and significant amount of research efforts have been dedicated to diagnosis and progression prediction of neurodegenerative diseases using different brain imaging modalities^3^,^18^,^19^,^22^,^25^. Automatic PD diagnosis and progression prediction could help physicians and patients avoid unnecessary medical examinations or therapies, as well as potential side effects and safety risks^26^. Machine learning and pattern recognition methods could simplify the development of these automatic PD diagnosis approaches. For instance, Prashanth *et al*.^18^ use intensity features extracted from SPECT images along with a SVM classifier, while Focke *et al*.^23^ use the voxel-based morphometry (VBM) on T1-weighted MRI with a SVM classifier to identify idiopathic Parkinson syndrome patients. In another work, Salvatore *et al*.^24^ propose a method based on principal component analysis (PCA) on morphological T1-weighted MRI, in combination with SVMs for diagnosis of PD and its syndromes.

Accordingly, the contributions of this paper are multi-fold: (1) Our proposed method selects the features that best classify the data in the kernel space, through learning multiple kernels; (2) We design kernel functions specific to our feature types (volumetric non-negative features); (3) We use multi-modal MRI and SPECT data and propose a non-linear feature fusion strategy to diagnose PD.

## PD Staging, Data Acquisition and Preprocessing

The data used in this paper are acquired from the PPMI database^9^, which is the first substantial study for identifying the PD progression biomarkers. In this research, we use the subjects with both MRI and SPECT modalities available in the database, resulting in 369 PD and 169 Normal Control (NC) subjects. PD subjects in the PPMI study are *de novo* PD patients, newly diagnosed and unmedicated. The healthy/normal control subjects are both age- and gender-matched with the PD patients. The demographic information of the subjects is illustrated in Table 1.

PD subjects are often evaluated using the widely used Hoehn and Yahr (H&Y) scale^27^ to understand their stagings. H&Y scale defines board categories, which rate the motor function for PD patients. H&Y stages correlate with several factors including motor decline, neuroimaging studies of dopaminergic loss and deterioration in quality of life^28^. It has a 5-point scale (Stages 1–5) measurement. Most of the studies in PD evaluated disease progression through analyzing patients and the time taken for them to reach one of the H&Y stages. The subjects in the first stage have unilateral involvement only, often with the least or no functional impairment. They have mild symptoms, which are inconvenient but not disabling. The second stage has bilateral or midline involvements, but still with no impairment of balance. For these subjects, the posture and gait are usually affected. Stage three shows the first signs of impaired reflexes. The patient will show significant slowing of the body movements and moderately severe dysfunction. In the fourth stage, the disease is fully developed and is severely disabling; the patient can still walk but to a limited extent, and might not be able to live alone any longer. In the fifth (final) stage, the patient will have a confinement to bed or will be bound to a wheelchair. The PD subjects in this study are mostly in the first two H&Y stages. As reported by the studies (http://www.ppmi-info.org/wp-content/uploads/2013/09/PPMI-WW-ADNI.pdf) in PPMI^9^, among the PD patients at the time of their baseline image acquisition, 43% of the subjects were in stage 1, 56% in stage 2 and the rest in stages 3 to 5.

**Figure 2:**
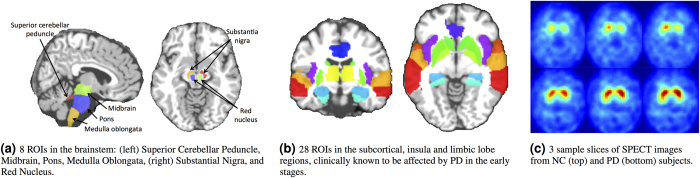
ROIs from MRI and SPECT used in this study.

Previous clinical studies (*e.g*., the Braak staging scheme^29^,^30^) show that the disease is initiated in brainstem, mid-brain and subcortical regions; and, over time, it propagates to many other brain regions. Thus, in order to diagnose PD and identify its imaging biomarkers in its *early stage*, we focus on the structures in the brainstem and basal ganglia areas, along with the regions in the subcortical regions. Based on^29^, we employ the 36 ROIs depicted in Fig. 2a, which correspond to brainstem (4 ROIs), substantia nigra and red nucleus (4 ROIs), limbic lobe (16 ROIs), insula (2 ROIs) and subcortical regions (10 ROIs). We extract features specifically from these ROIs, since we stress that we would like to investigate diagnosis of *de novo* PD subjects. Including all ROIs in the brain would jeopardize the motivation of early diagnosis, since other ROIs are previously investigated and are found not clinically relevant to PD or get affected by PD at very late stages^29^,^30^,^31^.

For each subject, a 3D MPRAGE sequence was performed, using 3T SIEMENS MAGNETOM TrioTim syngo scanners. T1-weighted images were acquired for 176 sagittal slices with the following parameters: repetition time = 2300 ms, echo time = 2.98 ms, flip angle = 9°, and voxel size = 1 × 1 × 1 mm^3^. The MR images are preprocessed by skull stripping, and then segmented into White Matter (WM), Gray Matter (GM), and CerebroSpinal Fluid (CSF) tissues, using technique in ref. 32. Then, the Anatomical Automatic Labeling (AAL) atlas with the above 36 labeled ROIs is registered to each subject’s native space. Specifically, we used FLIRT from FSL package^33^ for affine alignment, followed by HAMMER^34^ for nonlinear registration. The AAL atlas is warped according to the estimated deformation fields to the subject and then the ROI labels are propagated onto the subject’s native space. Finally, WM and GM tissue volumes in each ROI are calculated as MRI features.

On the other hand, SPECT images are primarily used for PD diagnosis and analysis. These images are used to register the dopamine transporter levels in the striatum, commonly known as DaTSCAN. To acquire this image, the _123_I-ioflupane neuroimaging radiopharmaceutical biomarker is injected, which binds to the dopamine transporters in the striatum. Brain images are then acquired. To process these images, the PPMI study (DatScan SPECT Image Processing Methods for Calculation of Striatal Binding Ratio, August 2013) has performed attenuation correction on the SPECT images, along with a standard 3D 6.0 mm Gaussian filter. Then, the images were normalized to standard Montreal Neurological Institute (MNI) space. Next, the transaxial slice with the highest striatal uptake was identified and the 8 hottest striatal slices around it were averaged, to generate a single slice image. On this average slice, the four caudate and putamen (left and right) ROIs, which are in the striatum brain region, are labeled and considered as target ROIs. The occipital cortex region is also segmented and used as a reference ROI. Count densities for the regions were used to calculate the striatal binding ratios (SBRs), as


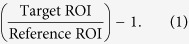


As can be seen in Fig. 2c, the caudate and putamen intensity values differ significantly between the PD and NC subjects.

## Notations

Throughout the paper, bold capital letters denote matrices (*e.g.*, **A**). Small bold letters are vectors (*e.g.*, **a**). All non-bold letters denote scalars or functions. 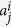 is the scalar in the row *i* and column *j* of **A**, while **a**^*i*^ and **a**_*j*_ denote the *i*^th^ row and the *j*^th^ column of **A**, respectively. 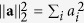 and 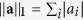 represent 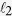 and 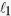 norms of **a**, respectively.

## Kernel-based Feature Selection and Max-Margin Classification

Feature or variable selection is defined as the process of picking a subset of discriminative features to best construct a model, namely for classification or regression. Feature selection approaches can be mainly categorized into two types: unsupervised and supervised. The former selects features without considering the class labels, while the latter aims to select features with the maximum relevance to the class labels and the least amount of redundancy in the selected features. Among the supervised feature selection methods, sparse feature selection^6^ has gained much attention in recent years, due to its simplicity and superior performance.

Suppose we have *N* training samples, with *d* features for each. We can arrange the feature vectors in a matrix 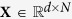, and their respective labels in 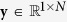. Sparse feature selection aims at minimizing the objective:


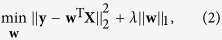


to best get a representation of the samples using the weight vector 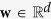. This weight vector is constrained with an 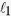 norm to get a compact set of discriminative features. Obviously, the set of selected features under this setting would be appropriate if we are planning to build a linear classification model (*e.g.*, linear SVM). This is because these features are selected to minimize redundancy and maximize relevance to the class labels in the original feature space. However, for a non-linear classification task, we should select the features that replicate the same minimum-redundancy and maximum-relevance in the kernel space.

Non-linear classification is usually achieved by first projecting the original feature vectors to a high-dimensional space using a non-linear mapping function (referred to as 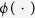), and then performing classification in the kernel space. However, instead of explicitly defining a mapping function, often a kernel function is used, which defines the similarity between the samples (or the feature vectors)^8^. This is simply because many machine learning algorithms (*e.g.*, SVMs) can be entirely expressed in terms of dot products^8^, and, under some conditions, kernel functions can also be expressed as dot products in a (possibly infinite dimensional) feature space.

Here, in order to both *select* features and *classify* the data, we adopt a formulation similar to kernel-based SVM, with a customized kernel to account for feature selection. We propose to apply the kernel function on each single feature and define the aggregate-kernel through a simple weighted sum of all these kernels. In this way, we can select the features that contribute most to constructing a better classification model through 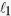 regularization on the kernels’ weight vector.

### Formulation

The original kernel-based SVM formulation finds a max-margin decision boundary in the kernel space,, using a loss function, *L*, with a trade-off hyperparameter *C*, as:


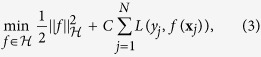


where *f* is associated with a Reproducing Kernel Hilbert Space 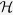, and **x**_*j*_ and *y*_*j*_ are the *j*^th^ sample and its corresponding label, respectively. In terms of a non-linear mapping function, *f* is defined as 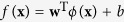. As discussed earlier, we do not directly use the mapping function, 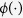. To this end, the representer’s theorem^35^ is incorporated, which states that the solution for **w** can always be represented as a linear combination of the training data. Therefore:


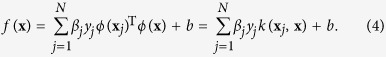


Hence, the optimization problem for the SVM would reduce to solving for *β*_*j*_, 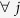.

Here, we would further like to select the features that can best construct the non-linear SVM model. Accordingly, we construct a single kernel for each feature, and then aggregate all these kernels through a weighted average. Therefore, the kernel between two samples **x**_*j*_ and **x** would be calculated as:


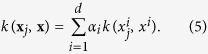


**Figure 3:**
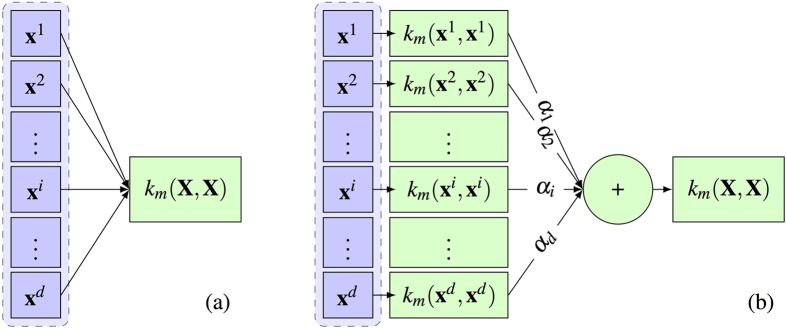
(**a**) Conventional construction of kernel functions. (**b**) Kernels learned for each single feature, aggregated through the weights corresponding to each feature. *k*_*m*_(.,.) denotes the kernel function applied on the features.

This is similar to multiple kernel learning frameworks^36^,^37^, while building each kernel only on a single feature. Applying the newly-introduced kernel *k* on all training samples, we would have **K** = *k*(**X**, **X**). Figure 3 shows the proposed process of building the kernel matrix over all samples (3b), in comparisons with the conventional kernel building (3a). Now, by regularizing the weight vector ***α***, we can enforce the selection of most discriminative features projected in the kernel space. Therefore, the objective function would be formed as:


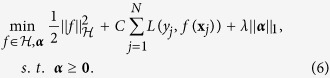


However, we know that usually different features and kernels characterize different aspects of the data. Therefore, we propose to use different kernel types on all features, and let our method choose which kernel type and which feature would best build the classification model. Accordingly, if we define *κ* different types of kernels, we define a combination of 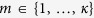 different kernels for the *d* features, as:


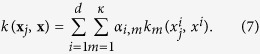


In this way, 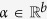 would be defined as the concatenation of all 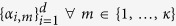. Note that *b* = *d* × *κ*. Since, there is a 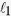 regularization on this vector, we can include different kernel types and determine which kernel contributes more in classifying the data, based on the selected feature(s).

### Optimization

To solve the optimization problem in (6), we only need to make sure the loss function is differentiable. We can, then, easily decompose the problem into two convex subproblems, and solve using a gradient decent method. The first subproblem would consist of solving for *f*, in which we assume ***α*** is fixed. This reduces to a simple SVM optimization with the kernel characterized by the fixed ***α***:


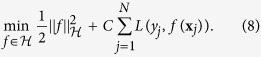



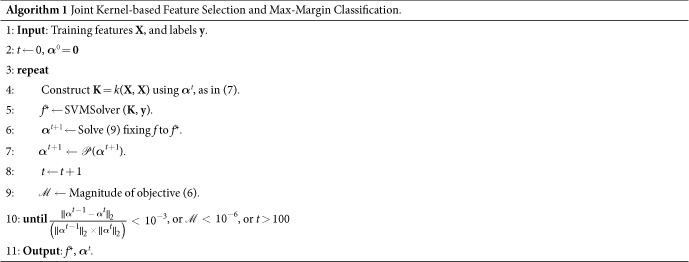


Then with *f* fixed, we can determine the best values for ***α*** through solving the second subproblem:


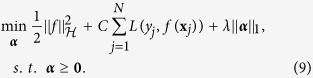


The only non-smooth term is the last one. However, since we need to enforce the constraint ***α*** ≥ 0, we use a projection operator 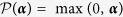 at each iteration. This constraint ensures that the last term is also differentiable with respect to ***α***. Therefore, this objective can be simply optimized using projected gradient decent^38^. To guarantee the convergence of the algorithm, based on^36^,^39^, we choose the gradient step size according to the Armijo rule^40^. As a result, the optimization process would alternate between the above two subproblems until convergence. The second subproblem learns the kernel, embedded in *f*(**x**_*j*_), through optimizing the weights ***α***, while the first one uses any SVM solver of choice to learn the SVM function.

Algorithm 1 summarizes the whole process and includes the stopping criteria. It is important to note that the main objective function (as in (6)) is a convex function, and therefore has a global optimum. The above two steps through the projected gradient decent will be repeated until convergence to the global optimum. It is shown that, under such settings with a convex objective function, the projected gradient decent converges to the global optimum^38^.

Note that our proposed framework for joint feature selection and classification integrates the 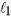 regularization on the max-margin classifier (*i.e.*, SVM), as in (6). Since the optimization process, as described above, consists of two phases of optimization, the same idea can be applied to other kernel methods, by using our customized kernel and incorporating the 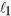 regularization to their formulations.

### Kernel Function

The choice of kernel functions is very important, dependent on the application and the data types associated with the specific application of interest. Kernels are basically a measure of similarity between the samples. One of the most popular kernels is the Radial Basis Function (RBF) or the Gaussian kernel, formulated as:


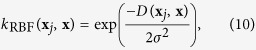


where *D*(.,.) is a distance measure between two samples and *σ* is the kernel hyperparameter. Usually a squared Euclidean norm is used as the distance measure between two samples:


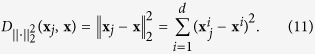


However, this distance metric should be adopted based on the characteristics of the data.

As described earlier, in this work, we use the volume of ROIs from MRI and SPECT images and therefore our features are all non-negative. Hence, our feature vectors are similar to histogram features or probability distribution functions (PDF), if normalized. Many previous works usually normalize the data using z-scores, which converts the feature values to a common scale with a mean of 0 and standard deviation of 1. However, this basically changes both the characteristics and the physical meanings of the features. But here, we normalize the feature vectors to the range [0, 1], making them similar to PDFs or the histogram-like features, used widely in computer vision applications. There are a number of other distance metrics used for non-negative or histogram-like features^41^. One of such distances is the *χ*^2^ distance:


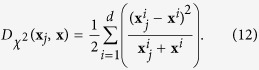


Another popular distance used for such purposes in the computer vision area is the Earth Mover’s Distance (EMD)^42^, which evaluates the dissimilarity between two multi-dimensional distributions in the feature space. Intuitively, given two distributions, we can consider one as a mass of earth spread in space, and the other as a collection of holes in that same space. EMD would measure the least amount of movements required to fill the holes with earth. For 1-dimensional case, such as the case for our application, EMD has a simple closed-form solution:


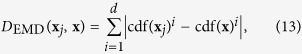


where cdf(⋅) is the cumulative distribution function and |·| is the absolute value function.

One of the popular kernels used for histogram-like features is the intersection kernel (histogram intersection kernel or HIK), which captures the similarities of the histogram-like features with the following definition:


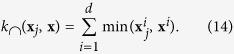


Since, dependent on the data distribution, some datasets might perform better using linear kernels (*i.e.*, in the native feature space), we also define the linear kernel function as:


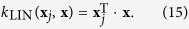


To find the best kernel, which can be different for each single dataset, we use all these kernel definitions and define our kernel formulation through Equation (7), by summing over all kernel types (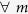, 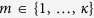), for each single feature (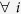, 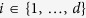). The set of all kernels could therefore be defined as the Linear kernel, the RBF kernel with different distance metrics, and the Intersection kernel as


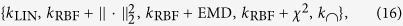


with *κ* = 5 different kernel types. Our optimization framework, through optimizing for *α*, will select which feature(s) and kernel type(s) are best for the problem with the available dataset. We will examine this proposed kernel and compare the results with different settings of the kernels and hyperparameters.

## Experimental Results

In this section, we conduct several experiments on synthetic and real PD diagnosis datasets. For evaluations, we compare our results with several baseline methods. Baseline classifiers under comparison include support vector machine (SVM), SparseSVM^43^, sparse feature selection followed by a SVM (SFS + SVM) and a multiple kernel learning (MKL) framework for SVM^37^, in which one kernel is learned for each modality and feature type (GM, WM and SBR, as described in Section) and classification is learned over these kernels. To further evaluate the effect of the feature selection (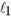 regularization), we run the same objective in (6) with conventional 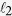 regularization on the vector *α*, denoted as ‘Proposed 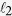-reg’. In addition, we also employ the following widely used linear and non-linear feature selection strategies followed by a SVM, and report the result: t-test^44^, elastic-net^45^, AutoEncoder-Restricted Boltzmann Machine (AE-RBM)^46^, and the mutual information based feature selector minimum-redundancy maximum-relevancy (mRMR)^7^. For all the experiments the hyperparameters are fixed, reported in the following. This avoids the excessive hyperparameter overturning procedures, and makes the results easily reproducible. All results are generated through 10-fold cross-validation, and we found that fixing *λ* = 1 generates the best results, while the best values for other hyperparameters are *σ* = 0.5 and *C* = 10.

### Synthetic Data

To verify the proposed method and analyze its performance, we generate two sets of synthetic data, one linearly separable and the other one not separable linearly (hence we believe it would be best classified nonlinearly). For both cases, the data comprise two classes of 100 samples each, and feature vectors of the dimensionality 50. The linearly separable data are sampled from two separate randomly generated subspaces. For the linearly separable experiment, we first construct two independent subspaces with the dimensionality of 50, similar to refs 2,47. These two subspaces are constructed with bases 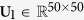 (a random orthogonal matrix), and 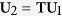, where **T** is a random rotation matrix. We then sample 50 vectors from each subspace through 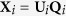, 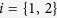, with **Q**_*i*_, a 50 × 100 matrix, being independent and identically distributed (*i.i.d*.) from 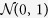. This leads to a binary classification problem. The two classes would correspond to the above two subspaces. For the nonlinearly separable data, we sample data for the two classes from two spheres in the 50-dimensional space, but with different radiuses. To this end, the first class data are sampled *i.i.d* from a sphere with the radius of 0.5. For the second class, we construct another sphere with a radius of 1.0 with the same center as the first sphere. Then, the data points of the second class are sampled *i.i.d* from the space comprised by the difference of the two spheres.

**Figure 4:**
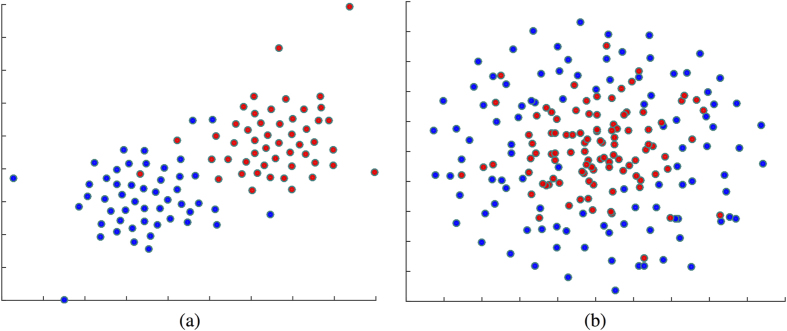
2D t-SNE projection of the synthetic data used for evaluation of the method. Red and Blue dots represent samples from two different classes. (**a**) Linearly separable data, (**b**) Nonlinearly separable data.

To sketch the data we generated for this experiments and to be able to visualize them, we employ a dimensionality reduction technique to facilitate the visualization of the data points. We project the samples from the two datasets into the 2D space using t-SNE^48^. The t-SNE projection technique visualizes high-dimensional data by giving each sample a location in a two dimensional map. The map created by the t-SNE reveals the neighborhood structure of the sample manifold at many different scales^48^. Figure 4 shows the 2D t-SNE projection of the generated data. As can be seen, the first set of data (4(a)) can be likely separated linearly, while the second one (4(b)) is not separable linearly, and we would expect to see better results using the non-linear kernels.

Each of the features for the two linear and nonlinear experiments are normalized to the range [0, 1], similar to the data we have, since our features from the neuroimaging data are volumetric and non-negative. The proposed method is run on these two sets of data, separately. The weight vector ***α***, which is defined in (7) and optimized in (6), will contain the weight for each feature (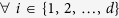) for all different *κ* kernel types (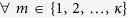). The mean weight for the features, across each kernel, would be a good indication of how the useful kernels for the task of classification were selected. These mean values are included in Table 2. Since we have imposed a 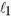 regularization on the weights vector, it will sparsely select a compact set of features and kernels. Therefore, features with the less-useful or redundant kernels are often all given zero weights. As can be seen, for the linearly separable data (Fig. 4(a)), the linear and histogram intersection kernels are selected, while for the non-linear data, the RBF kernel with different distance metrics are selected along with the HIK. One of the main conclusions here could be that HIK is almost equally very important for both linear and non-linear volumetric data. We will verify this on the real neuroimaging dataset, in the following subsections. HIK kernel has also been found to very useful in the computer vision area, in which the features are non-negative and histogram-like. This type of kernel can model non-linear relations, while being also able to model piece-wise linear problems^49^. This is the reason it is found very useful for both linear and non-linear problems.

It is noteworthy to mention that our method can work equally the same for any type linearly or non-linearly separable data. In this case, the method for both experiments, generates a 100% accuracy, while based on the characteristics of the data, different kernel types are selected.

### PD Diagnosis

As described before, we use both MRI and SPECT modalities of total 538 subjects (169 NC, 369 PD) from the PPMI database to evaluate the proposed method. Table 3 compares the diagnosis accuracies of the proposed and baseline methods, analyzing the effects of different modalities. As it is obvious, the proposed method generates the best results when using both MRI and SPECT modalities. Additionally, Table 4 shows the results of the proposed and the baseline kernel methods with different kernel settings using the MRI + SPECT modalities. The proposed kernel, which combines all kernel types provides the best results compared to the other five kernels. Our method (Proposed 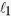-reg and 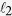-reg) is the only method that can interweave all kernels and concurrently select the best kernel and feature combination. Other kernel methods (MKL, SFS + SVM and SVM, as listed in the Table) can only operate on a single kernel and hence would only be able to interpret the data from a single aspect (*e.g.*, linearity or non-linearity).

**Figure 5:**
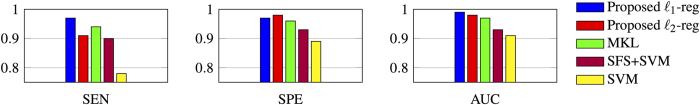
Comparisons of the sensitivity (SEN), specificity (SPE) and area under the ROC curve (AUC).

In addition, Fig. 5 compares the sensitivity, specificity and the area under ROC curve for the methods. As can be seen, the proposed framework using the proposed interweaved kernel on the combination of SPECT and MRI data achieves the best results with an accuracy of 97.5%. This suggests that the best features are selected in the non-linear kernel space, compared to the results of other methods, specifically SFS + SVM with an accuracy of 90.8%, in which a sparse feature selection is conducted in the original feature space followed by a non-linear classifier. This supports our assumption that, if a non-linear classification is intended, we should select those features that form the best classifier in the kernel space, not in the original feature space. Furthermore, our proposed kernel achieves an improved accuracy of over 1.4% than the case of squared Euclidean distance, which is widely used in many previous studies. On the other hand, comparison of the results from 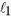 and 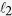 regularization of the kernel weights vector indicates that the proposed method (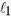 regularized) achieves an accuracy (and AUC) superior to the same formulation but with 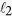 regularization. This is because 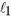 regularization is suitable for recovering sparse signals, and can certainly reduce overfitting and increase generalization capability, thus achieving better performance on the unseen testing data. 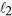 is preferable for data that is not sparse, while our features are extracted from brain ROIs. 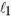 enforces selecting most discriminant ROIs. To further evaluate the feature selection performance, we conducted experiments with several state-of-the-art linear and non-linear feature selection or reduction and classification techniques used for such neurodegenerative diagnosis applications, which are already included in Table 3.

To analyze the effect of the proposed kernel and the contribution of each single kernel in building the final max-margin classification model, we calculate the mean weight (in vector ***α***) of the features for each single kernel. Therefore, for the *m*^th^ kernel this mean weight would be calculated as


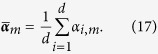


**Figure 6:**
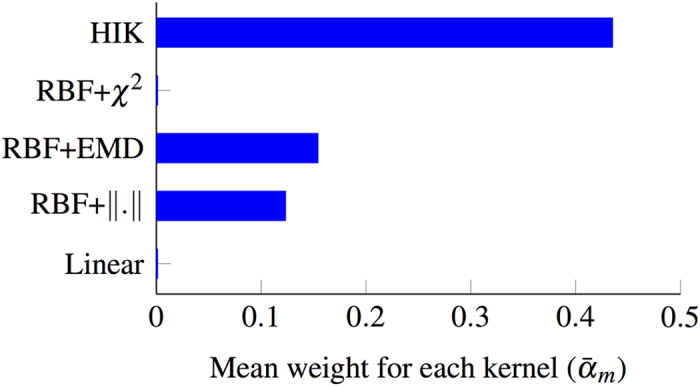
Mean weight of the features for each of the kernels, selected by our method. The larger the mean weight, the more frequently that kernel is selected with larger weights, and thus more useful it can be.

**Figure 7:**
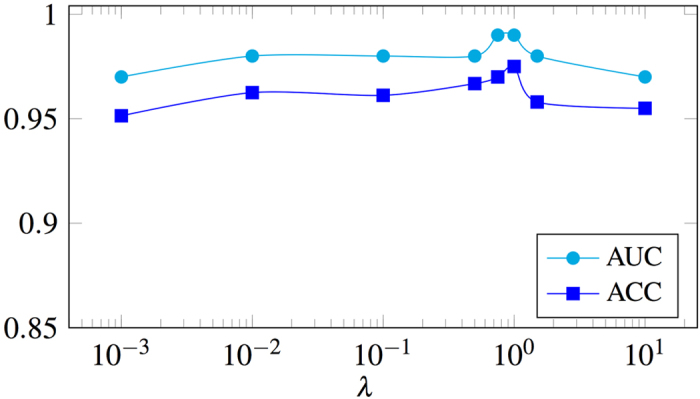
Accuracy (ACC) and area under the ROC curve (AUC) as a function of the hyperparameter *λ*, as in (6).

This measure can simply show how often features calculated using that kernel are selected and therefore would indicate the importance and significance of that kernel type. Figure 6 shows these mean weight values. As can be seen, again the intersection kernel (HIK) has the largest weight while the other two variations of RBF kernel (*i.e.*, with EMD and Euclidian distance) are getting less weights, but still important. The other two kernels, linear and RBF with the *χ*^2^ distance, however, are not showing any useful for the task at hand. This is aligned with the non-linear synthetic experiment and shows that if the features selected according to the classifier the performance could be boosted. To analyze the hyperparameter, *λ* in the objective function (6), we plot the accuracy and the area under the ROC curve as a function of *λ* in Fig. 7. This hyperparameter is a trade-off to control how compact we want the selected feature set to be. As it is clear in this figure, the whole process is not hugely dependent to the setting of this hyperparameter and performs similarly well in a wide range of its settings.

**Figure 8:**
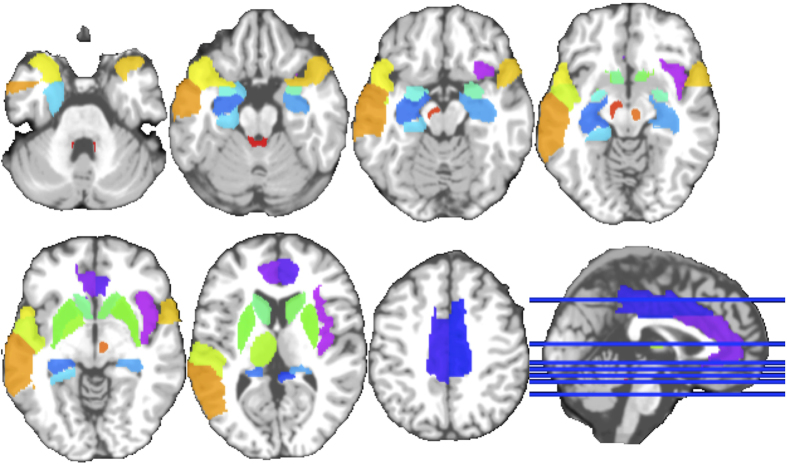
The most frequently selected ROIs.

One the major advantages of the proposed technique is that we can analyze the weight vector ***α*** and see features corresponding to which ROI are given non-zero mean weights over the 10 folds of the cross-validation. In this way, we can draw a conclusion on the importance of any single ROI for the specific disease. The selected ROIs by the proposed method are visualized in Fig. 8, and Table 5 depicts the specific ROIs and tissue types or modalities that were selected. The last row of this table shows the mean values for the coefficient vector ***α*** for that specific modality (feature type), which indicates the level of significance and contribution of specific feature types in building the kernel-based max-margin classifier. The SPECT features are the most useful features as they are quite discriminative for the task of PD diagnosis. This is also easily inferred by comparing the two subject samples in Fig. 2c. Among the MRI features, WM volumes are more compelling, since the deep brain regions contain more WM volumes and fibers. The selected ROIs are also aligned with the previous studies^2^,^20^,^29^,^30^,^50^. Specifically, the Putamen and Caudate regions, along with the brainstem and many subcortical regions have always been studied and found important to assess PD development measures.

**Figure 9:**
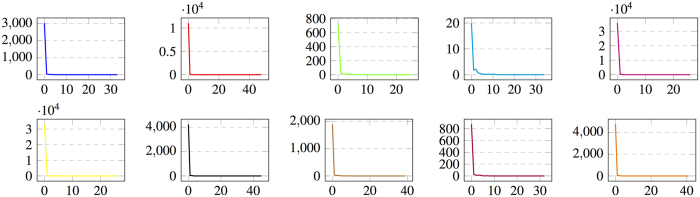
The magnitude of objective function in (6) (denoted as 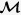 in Algorithm 1), as a function of the number of iterations required for solving the alternating optimization problem, over all 10 folds of the cross-validation experiment.

To analyze the convergence behavior of the proposed method, we plot the magnitude of the objective function in (6) for each of the 10 folds in the experiment on PPMI using both MRI and SPECT modalities, as a function of the number of iterations required for solving the alternating optimization problem. The plots are illustrated in Fig. 9. As can be seen, the algorithm converges in a limited number of iterations for all data splits.

**Figure 10:**
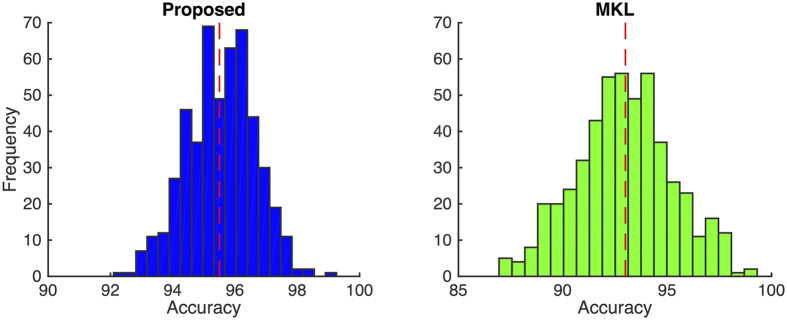
Histograms of bootstrapping for 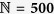 different trials, for the proposed (with 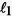-regularization) and MKL methods. Red dashed line indicates the mean accuracy for all the trials. Our proposed method has a better mean accuracy with a tighter bound for the standard deviation.

To analyze the significance of the obtained results, we conduct a permutation test^51^, which does not assume any data distribution and is also non-parametric. Permutation test investigates whether the classifier has found any class structure, or the observed accuracy was obtained by coincidence. To this end, the classification scheme is repeated by randomly permuting the class labels for *π* different times (*π* = 100, in our experiments). Then, a *p*-value can be calculated, which would show the portion of the runs for which the misclassification rate is better than the original classification error. For our case, we obtained a *p*-value less than 0.01, which signifies that the classification error on the original data is indeed substantially small and the classifier is not generating those results by chance^51^. Furthermore, we use a bootstrapping procedure to statistically validate the performance of the proposed method. In this way, we can alternatively show that the obtained results are not due to any over-fitting. But we only have one dataset. As a result, when we compute a statistic on the data (*i.e.*, classification accuracy for our case), we cannot see how variable that statistic is. Accordingly, we create a large number of datasets, through bootstrapping, by subsampling from the original dataset. This leads to a distribution for the accuracy, so we can compare the methods statistically. To this end, we resample 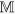 subjects *with replacement* from the dataset. Then, we use 90% of these subjects to train the classifier, which is then used to classify the remaining 10% of the subjects. This procedure is repeated 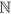 different times, leading to 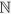 different accuracy results. For our case, we set 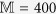 and 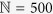. Since the subjects are sampled with replacement, the trials are independent and identically distributed (*i.i.d*.). Therefore, we can compare the methods using their 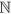 trial bootstrap performances. Figure 10 shows the histograms of the 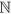 trials for our proposed method (with 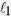-regularization) and the baseline MKL method. With all the trials, our proposed method and the baseline MKL method achieved the mean and standard deviation of 95.5% ± 1.1 and 93.0 ± 2.2, respectively. As can be seen, our proposed method has a better mean accuracy with a tighter bound for the standard deviation, compared to the baseline MKL method.

In addition, we compare our proposed method against several previously published state-of-the-art methods for the same purpose (PD diagnosis). The comparisons are provided in Table 6. The table includes all the information about the dataset and the methods they used for obtaining those results. As can be seen, our results are superior to all previous works, while we evaluated our method on a large dataset. One important note here could be that MRI data are much less dependable for the diagnosis task. This can be why when a small dataset is utilized, the solutions are quite prone to overfitting and relatively reasonable results could be achieved. On the other hand, when using large sets of data, the results for the diagnosis using only MRI degrades. Furthermore, the comparisons with the work of Prashanth *et al*.^18^ shows that the combination of MRI and SPECT data boosts the performance of diagnosis. It is important to note that the experiments in ref. 18 are conducted on the same PPMI dataset, but the authors used subjects from multiple time points, putting them together and running their method. This could be prone to several issues: (1) the subjects in the future time points, which were diagnosed as PD in the previous time points, are now in the late stages of PD, and (2) different time points of the same subject can be split in the training and testing sets, which helps unfairly increase the performance. Although the comparison is not fair, still we outperform their results by combining multiple modalities and proposing a more robust method.

## Conclusion

In this paper, we introduced a kernel-based feature selection scheme, in which we select features and kernels that induce the best classification performance in the kernel space. Furthermore, we presented a kernel function by interweaving different kernel types, which is in accordance with the feature types, while being able to tolerate both linear and non-linear properties of the data. The results indicate that the proposed framework for joint kernel-based feature selection and max-margin classification induces the best performance for diagnosis for Parkinson’s disease, when using features from both SPECT and MRI modalities. We tested the proposed method on a large set of data and obtained competitive and superior results for the diagnosis of PD among all previous studies for PD.

## Additional Information

**How to cite this article:** Adeli, E. *et al*. Kernel-based Joint Feature Selection and Max-Margin Classification for Early Diagnosis of Parkinson’s Disease. *Sci. Rep.* **7**, 41069; doi: 10.1038/srep41069 (2017).

**Publisher's note:** Springer Nature remains neutral with regard to jurisdictional claims in published maps and institutional affiliations.

## Figures and Tables

**Figure 1:**
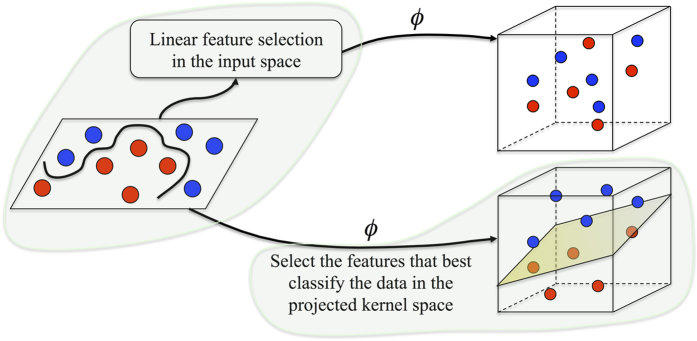
Conventional methods (*e.g*., refs 3 and 7) usually select features in the original input feature space and then learn a classifier based on the selected features (Top). However, if a non-linear classification is intended, it is better to select the features that can best classify the data in the kernel space (Bottom). Here, 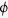 denotes a non-linear mapping function.

**Table 1:** Details of the subjects from the PPMI dataset used in our study.

	Total	Gender		Age (years)	Edu. (years)
		F	M		
PD	369	131	238	61.50 ± 9.62	15.58 ± 2.93
NC	169	59	110	60.42 ± 11.43	16.09 ± 2.87

‘Age’ indicates the mean ± standard deviation (std) of the subject ages (in years) in that category. Similarly, ‘Edu.’ denotes the mean ± std of the amount of education (in years) of the subjects.

**Table 2:** Results on the synthetic data: mean weight of the features for each of the *κ* = 5 kernels.

Data	Linear	RBF + ||·||	RBF + EMD	RBF + *χ*^2^	HIK
Fig. 4(a)	0.298	0	0	0	0.539
Fig. 4(b)	0	0.227	0.254	0	0.445

**Table 3:** Diagnosis accuracy of the proposed and the baseline methods, with different modalities.

Method	MRI	SPECT	MRI + SPECT
Proposed 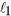 -reg	70.5%	95.6%	**97**.**5**%
Proposed 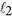 -reg	69.8%	95.4%	95.5%
MKL	N/A	N/A	95.7%
SFS + SVM	60.1%	94.1%	90.8%
SVM	56.2%	94.0%	85.8%
t-test + SVM	59.1%	93.5%	91.2%
elastic-net + SVM	60.1%	94.1%	93.5%
AE-RBM + SVM	62.7%	94.5%	96.1%
mRMR + SVM	59.9%	93.9%	92.1%
SparseSVM	58.5%	93.6%	93.8%

**Table 4:** Diagnosis accuracy of the proposed and the baseline methods, with different kernels on MRI + SPECT data.

Method	Proposed, as in (16)	RBF + EMD	RBF + *χ*^2^	RBF + 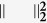	HIK	Linear
Proposed 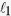 -reg	97.5%	96.8%	95.8%	96.1%	96.9%	93.9%
Proposed 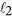 -reg	95.8%	95.5%	94.9%	95.8%	94.9%	93.1%
MKL	N/A	95.7%	95.1%	95.4%	96.5%	93.2%
SFS + SVM	N/A	89.8%	88.5%	90.7%	90.8%	87.9%
SVM	N/A	85.8%	85.1%	85.5%	84.9%	84.8%

**Table 5:** ROI names and their modalities, selected as the most important ROIs by our algorithm.

Region of Interest (ROI)	WM	GM	SBR
Insula left			
Insula right	✓	✓	
Anterior cingulate gyrus left		✓	
Anterior cingulate gyrus right		✓	
Middle cingulate gyrus left	✓	✓	
Middle cingulate gyrus right	✓	✓	
Posterior cingulate gyrus left			
Posterior cingulate gyrus right		✓	
Hippocampus left		✓	
Hippocampus right	✓	✓	
ParaHippocampal gyrus left	✓	✓	
ParaHippocampal gyrus right			
Amygdala left		✓	
Amygdala right		✓	
Caudate left			✓
Caudate right	✓		✓
Putamen left			✓
Putamen right	✓	✓	✓
Pallidum left	✓		
Pallidum right			
Thalamus left		✓	
Thalamus right			
Superior temporal gyrus left	✓		
Superior temporal gyrus right			
Temporal pole (superior) left	✓	✓	
Temporal pole (superior) right		✓	
Middle temporal gyrus left	✓		
Middle temporal gyrus right		✓	
Midbrain			
Pons			
Medulla oblongata			
Superior cerebellar peduncle	✓	✓	
Substantia nigra Left	✓		
Substantia nigra Right			
Red nucleus Left			
Red nucleus Right	✓		
Mean value of coefficients	0.2365	0.2131	1.6223

**Table 6:** Comparisons of the proposed method with state-of-the-art methods for PD diagnosis.

Method	Subjects		Methodology	Modalities	PD *vs*. NC (%)
	PD	NC			
Prashanth^18^	493	181	Linear Classifier	SPECT	92.3
Prashanth^18^	493	181	Non-linear Classifier	SPECT	96.1
Salvatore^31^	28	28	PCA + SVM	MRI	85.8
Adeli-M^2^	56	56	Robust Feature Sample LDA	MRI	84.1
**Ours**	369	169	Kernel-based Feature Selection and Max-Margin Classification	MRI + SPECT	**97**.**5**

The table includes number of subjects and the methodologies used by different methods. Also, the neuroimaging modalities used by each study are provided.

## References

[b1] Yuan L., Wang Y., Thompson P. M., Narayan V. A. & Ye J. Multi-source feature learning for joint analysis of incomplete multiple heterogeneous neuroimaging data. NeuroImage 61, 622–632 (2012). 22498655 10.1016/j.neuroimage.2012.03.059 PMC3358419

[b2] Adeli-Mosabbeb E., Thung K.-H., An L., Shi F. & Shen D. Robust feature-sample linear discriminant analysis for brain disorders diagnosis. In NIPS (2015).

[b3] Thung K.-H., Wee C.-Y., Yap P.-T., Shen D. & Initiative A. D. N. Neurodegenerative disease diagnosis using incomplete multi-modality data via matrix shrinkage and completion. NeuroImage 91, 386–400 (2014). 24480301 10.1016/j.neuroimage.2014.01.033 PMC4096013

[b4] Kerr W. T. . Parameter selection in mutual information-based feature selection in automated diagnosis of multiple epilepsies using scalp EEG. In PRNI (2012). 10.1109/PRNI.2012.27 PMC4169072 25241830

[b5] Gu Q., Li Z. & Han J. Generalized fisher score for feature selection. In UAI (2011).

[b6] Wright J., Yang A. Y., Ganesh A., Sastry S. S. & Ma Y. Robust face recognition via sparse representation. IEEE TPAMI 31, 210–227 (2009). 10.1109/TPAMI.2008.79 19110489

[b7] Peng H., Long F. & Ding C. Feature selection based on mutual information criteria of max-dependency, max-relevance, and min-redundancy. IEEE TPAMI 27, 1226–1238 (2005). 10.1109/TPAMI.2005.159 16119262

[b8] Hofmann T., Schölkopf B. & Smola A. J. Kernel methods in machine learning. Ann. Stat. 1171–1220 (2008).

[b9] Marek K. . The parkinson progression marker initiative (PPMI). Progress in Neurobiology 95, 629–635 (2011). 21930184 10.1016/j.pneurobio.2011.09.005 PMC9014725

[b10] Hastie T., Tibshirani R. & Friedman J. The elements of statistical learning: data mining, inference and prediction, 2 edn. (Springer, 2009).

[b11] Garrett D., Peterson D. A., Anderson C. W. & Thaut M. H. Comparison of linear, nonlinear, and feature selection methods for EEG signal classification. IEEE TNSRE 11, 141–144 (2003). 10.1109/TNSRE.2003.814441 12899257

[b12] Liu M., Zhang D., Adeli E. & Shen D. Inherent structure-based multiview learning with multitemplate feature representation for alzheimer’s disease diagnosis. IEEE TBME 63, 1473–1482 (2016). 10.1109/TBME.2015.2496233 PMC4851920 26540666

[b13] Rondina J. M. . SCoRS - a method based on stability for feature selection and mapping in neuroimaging. IEEE TMI 33, 85–98 (2014). 10.1109/TMI.2013.2281398 PMC4576737 24043373

[b14] Tohka J., Moradi E., Huttunen H. & ADNI. Comparison of feature selection techniques in machine learning for anatomical brain mri in dementia. Neuroinformatics 1–18 (2016). 26803769 10.1007/s12021-015-9292-3

[b15] Jankovic J. Parkinson’s disease: clinical features and diagnosis. Journal of Neurology, Neurosurgery & Psychiatry 79, 368–376 (2008). 10.1136/jnnp.2007.131045 18344392

[b16] Menke R. A. . MRI characteristics of the substantia nigra in parkinson’s disease: A combined quantitative T1 and DTI study. NeuroImage 47, 435–441 (2009). 19447183 10.1016/j.neuroimage.2009.05.017

[b17] Loane C. & Politis M. Positron emission tomography neuroimaging in parkinson’s disease. American Journal of Translational Research 3, 323–341 (2011). 21904653 PMC3158735

[b18] Prashanth R., Roy S. D., Mandal P. K. & Ghosh S. Automatic classification and prediction models for early parkinson’s disease diagnosis from SPECT imaging. Expert Syst. Appl. 41, 3333–3342 (2014).

[b19] Duchesne S., Rolland Y. & Varin M. Automated computer differential classification in parkinsonian syndromes via pattern analysis on MRI. A. Radiology 16, 61–70 (2009). 10.1016/j.acra.2008.05.024 19064213

[b20] Adeli E. . Joint feature-sample selection and robust diagnosis of parkinson’s disease from MRI data. NeuroImage 141, 206–219 (2016). 27296013 10.1016/j.neuroimage.2016.05.054 PMC5866718

[b21] Ziegler D. & Augustinack J. Harnessing advances in structural MRI to enhance research on Parkinson’s disease. Imag. in med. 5, 91–94 (2013). 10.2217/iim.13.8 PMC3655710 23687517

[b22] Marquand A. . Automated, high accuracy classification of parkinsonian disorders: a pattern recognition approach. PLoS One 8, e69237 (2013). 23869237 10.1371/journal.pone.0069237 PMC3711905

[b23] Focke N. K. . Individual voxel-based subtype prediction can differentiate progressive supranuclear palsy from idiopathic parkinson syndrome and healthy controls. Human Brain Mapping 32, 1905–1915 (2011). 21246668 10.1002/hbm.21161 PMC6870106

[b24] Salvatore C. . Machine learning on brain MRI data for differential diagnosis of parkinson’s disease and progressive supranuclear palsy. Journal of Neuroscience Methods 222, 230–237 (2014). 24286700 10.1016/j.jneumeth.2013.11.016

[b25] Rizk-Jackson A. . Evaluating imaging biomarkers for neurodegeneration in pre-symptomatic huntington’s disease using machine learning techniques. NeuroImage 56, 788–796 (2011). 20451620 10.1016/j.neuroimage.2010.04.273

[b26] Cummings J. L. . The role of dopaminergic imaging in patients with symptoms of dopaminergic system neurodegeneration. Brain 134, 3146–3166 (2011). 21810889 10.1093/brain/awr177

[b27] Hoehn M. & Yahr M. Parkinsonism: onset, progression and mortality. Neurology 17, 427–442 (1967). 6067254 10.1212/wnl.17.5.427

[b28] Bhidayasiri R. & Tarsy D. Movement Disorders: A Video Atlas (Springer, 2012).

[b29] Braak H. . Staging of brain pathology related to sporadic parkinson’s disease. Neurobio. of Aging 24, 197–211 (2003). 10.1016/s0197-4580(02)00065-9 12498954

[b30] Burke R. E., Dauer W. T. & Vonsattel J. P. G. A critical evaluation of the braak staging scheme for parkinson’s disease. Annals of neurology 64, 485–491 (2008). 19067353 10.1002/ana.21541 PMC2605160

[b31] Salvatore C. . Machine learning on brain MRI data for differential diagnosis of Parkinson’s disease and progressive supranuclear palsy. J. of neuroscience methods 222, 230–237 (2014). 10.1016/j.jneumeth.2013.11.016 24286700

[b32] Pham D. L. & Prince J. L. An adaptive fuzzy c-means algorithm for image segmentation in the presence of intensity inhomogeneities. Pattern recognition letters 20, 57–68 (1999).

[b33] Jenkinson M., Bannister P., Brady M. & Smith S. Improved optimization for the robust and accurate linear registration and motion correction of brain images. Neuroimage 17, 825–841 (2002). 12377157 10.1016/s1053-8119(02)91132-8

[b34] Wu G., Yap P.-T., Kim M. & Shen D. TPS-HAMMER: Improving HAMMER registration algorithm by soft correspondence matching and thin-plate splines based deformation interpolation. NeuroImage 49, 2225–2233 (2010). 19878724 10.1016/j.neuroimage.2009.10.065 PMC2818392

[b35] Kimeldorf G. S. & Wahba G. A correspondence between bayesian estimation on stochastic processes and smoothing by splines. Ann. Math. Stat. 495–502 (1970).

[b36] Varma M. & Babu B. R. More generality in efficient multiple kernel learning. In ICML, 1065–1072 (2009).

[b37] Saghafi B., Rajan D. & Li W. Efficient 2d viewpoint combination for human action recognition. Pattern Analysis and Applications 19, 563–577 (2016).

[b38] Calamai P. H. & Moré J. J. Projected gradient methods for linearly constrained problems. Mathematical programming 39, 93–116 (1987).

[b39] Chapelle O., Vapnik V., Bousquet O. & Mukherjee S. Choosing multiple parameters for support vector machines. Machine learning 46, 131–159 (2002).

[b40] Liu P.-L. & Der Kiureghian A. Optimization algorithms for structural reliability. Structural safety 9, 161–177 (1991).

[b41] Cabral R. S., De la Torre F., Costeira J. P. & Bernardino A. Matrix completion for weakly-supervised multi-label image classification. IEEE TPAMI (2015). 10.1109/TPAMI.2014.2343234 26353213

[b42] Ling H. & Okada K. An efficient earth mover’s distance algorithm for robust histogram comparison. IEEE TPAMI 29, 840–853 (2007). 10.1109/TPAMI.2007.1058 17356203

[b43] Tan M., Wang L. & Tsang I. W. Learning sparse SVM for feature selection on very high dimensional datasets. In ICML, 1047–1054 (2010).

[b44] Guyon I. & Elisseeff A. An introduction to variable and feature selection. JMLR 3, 1157–1182 (2003).

[b45] Zou H. & Hastie T. Regularization and variable selection via the elastic net. J. of Royal Statistical Society: Series B (Statistical Methodology) 67, 301–320 (2005).

[b46] Salakhutdinov R., Mnih A. & Hinton G. Restricted boltzmann machines for collaborative filtering. In ICML, 791–798 (2007).

[b47] Liu G. . Robust recovery of subspace structures by low-rank representation. IEEE TPAMI 35, 171–184 (2013). 10.1109/TPAMI.2012.88 22487984

[b48] Van der Maaten L. & Hinton G. Visualizing data using t-SNE. JMLR 9, 85 (2008).

[b49] Maji S., Berg A. C. & Malik J. Classification using intersection kernel support vector machines is efficient. In CVPR (2008).

[b50] Worker A. Cortical thickness, surface area and volume measures in parkinson’s disease, multiple system atrophy and progressive supranuclear palsy. PLoS One 9 (2014). 10.1371/journal.pone.0114167 PMC4252086 25463618

[b51] Ojala M. & Garriga G. C. Permutation tests for studying classifier performance. The Journal of Machine Learning Research 11, 1833–1863 (2010).

